# No3CoGP: non-conserved and conserved coexpressed gene pairs

**DOI:** 10.1186/1756-0500-7-886

**Published:** 2014-12-08

**Authors:** Chittabrata Mal, Md Aftabuddin, Sudip Kundu

**Affiliations:** Department of Biophysics, Molecular Biology & Bioinformatics, University of Calcutta, 92, A.P.C. Road, Kolkata, 700009 India; West Bengal University of Technology, BF-142, Salt Lake, Sector I, Kolkata, 700064 India; Center of Excellence in Systems Biology and Biomedical Engineering (TEQIP Phase II), University of Calcutta, Kolkata, India

**Keywords:** Gene coexpression, Affymetrix microarray, Differential coexpression, Gene modules

## Abstract

**Background:**

Analyzing the microarray data of different conditions, one can identify the conserved and condition-specific genes and gene modules, and thus can infer the underlying cellular activities. All the available tools based on Bioconductor and R packages differ in how they extract differential coexpression and at what level they study. There is a need for a user-friendly, flexible tool which can start analysis using raw or preprocessed microarray data and can report different levels of useful information.

**Findings:**

We present a GUI software, No3CoGP: Non-Conserved and Conserved Coexpressed Gene Pairs which takes Affymetrix microarray data (.CEL files or log2 normalized.txt files) along with annotation file (.csv file), Chip Definition File (CDF file) and probe file as inputs, utilizes the concept of network density cut-off and Fisher’s z-test to extract biologically relevant information. It can identify four possible types of gene pairs based on their coexpression relationships. These are (i) gene pair showing coexpression in one condition but not in the other, (ii) gene pair which is positively coexpressed in one condition but negatively coexpressed in the other condition, (iii) positively and (iv) negatively coexpressed in both the conditions. Further, it can generate modules of coexpressed genes.

**Conclusion:**

Easy-to-use GUI interface enables researchers without knowledge in R language to use No3CoGP. Utilization of one or more CPU cores, depending on the availability, speeds up the program. The output files stored in the respective directories under the user-defined project offer the researchers to unravel condition-specific functionalities of gene, gene sets or modules.

**Electronic supplementary material:**

The online version of this article (doi:10.1186/1756-0500-7-886) contains supplementary material, which is available to authorized users.

## Findings

### Background

Analysis of differential expression of a gene, coexpression of gene pairs and set of genes in one condition (class) compared to the other condition helps the researchers to unravel condition-specific functionalities of gene, gene sets or modules [1]. Some of the important biological phenomena which can be unravelled from this analysis, include the loss of function of a protein complex, identifying functional gene modules in a diseased condition [2] and the combinatorial regulation of genes [3] etc. Several algorithms and software, available for this purpose, mainly vary in how those quantify coexpression [reviewed in [4]], differential coexpression [5] and what biological questions those want to address. While some detect the coexpressed gene pairs using a measure of correlation (e.g., Pearson Correlation Coefficient), others like ARACNE [6] and CLR [7] use mutual information theory. Among the tools used to identify differential coexpression, DICER can identify the set of genes showing significantly higher coexpression in one class than other [8]. Very recently developed C3D can detect both common and condition specific clusters based on sophisticated statistical algorithm; however it is MATLAB dependent [9]. Within the R-packages, while the previous version of DCGL can differentiate significant changes in coexpression among different conditions, the latest version (DCGL 2.0) can identify the differential regulation [3].

Here, we have developed a user friendly, flexible, standalone software, No3CoGP which takes Affymetrix microarray data (.CEL files or log2 normalized.txt files) along with annotation file (.csv file), Chip Definition File (CDF file) and probe file as inputs and can identify four possible types of gene pairs based on their coexpression relationships. To extract the biologically significant gene pairs, we have implemented different levels of filterings including the concept of network density cut-off suggested by Aoki et al. [10]. Among the four possible types of gene pairs two are non-conserved gene pairs - (i) showing coexpression in one class but not in other (differentially coexpressed non-conserved gene pair, DCNCGP), (ii) positively coexpressed in one class but negatively coexpressed in the other class (contra-coexpressed non-conserved gene pair, CCNCGP); other two are conserved gene pairs - (iii) positively coexpressed in both the classes (positively coexpressed conserved gene pair, PCCGP) and (iv) negatively coexpressed in both the classes (negatively coexpressed conserved gene pair, NCCGP). Finally, modules of different types of coexpressed genes can be generated.

### Implementation

**Figure 1:**
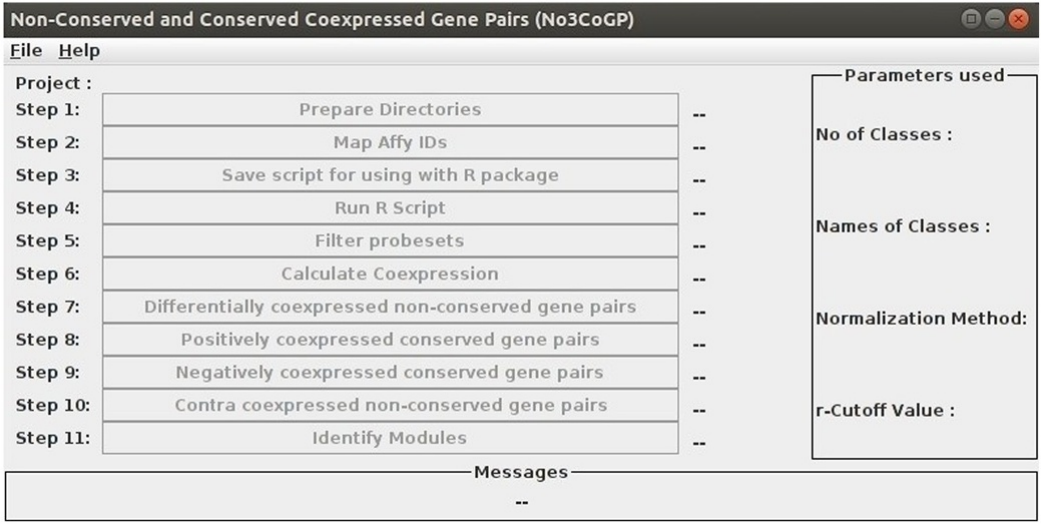
**No3CoGP GUI.** The GUI consists of top menu bar, project steps, job status, parameters used box and message box.

**Figure 2:**
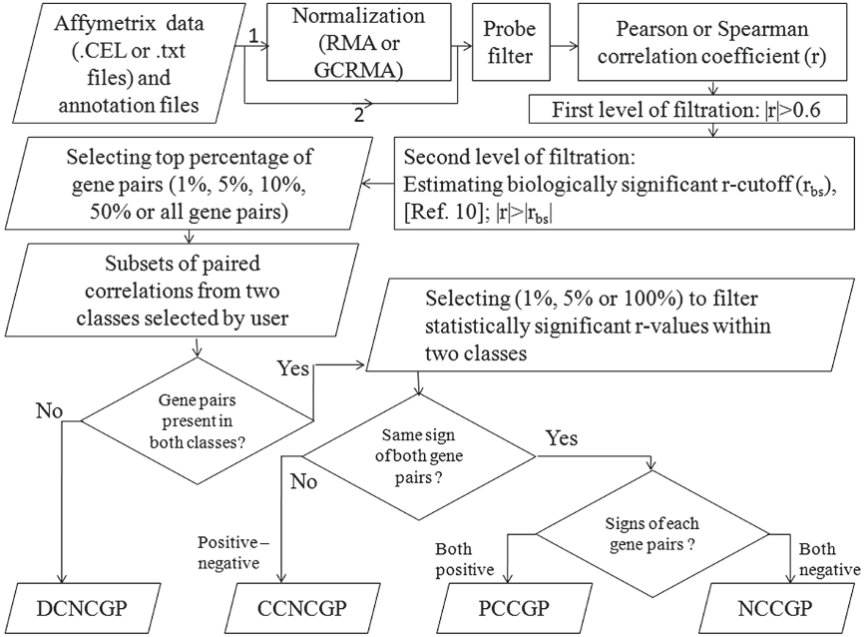
**Flowchart.** Flowchart to identify different types of coexpressed gene pairs. 1 indicates the path when raw microarray data files are given as input and 2 indicates the path when log2 normalized files are given as input.

The Java-based program has been written to utilize one or more CPU cores available during extensive calculations and tested in LINUX and Windows environments. However, run-time will depend on the size of input data. The GUI consists of top menu bar, project steps, job status, parameters used box and message box (Figure 1). In the console the user can view various parameters, status and results. Since the computational algorithm of No3CoGP is layered into multiple steps, the user can halt the process after the completion of any intermediate step and can resume the job later. The work-flow of the software is given in Figure 2.

### Results and discussion

The functionality and applicability of No3CoGP have been expedited in a case study. We have tested and analyzed microarray data (samples from series GSE19326) of three tomato tissues (e.g., leaf, peel and flesh) to find out conserved and non-conserved gene pairs. Using ‘UniGene’ as annotation id, ‘GCRMA’ as normalization method and Pearson Correlation as correlation measure we have found several gene pairs and modules at different significant levels (see Additional file 1. Coexpressed gene pairs and modules). Further analysis is required to identify the significant relationships among the genes present in the modules and to understand the underlying principle of gene regulation.

Any tool analyzing the co-expression of larger microarray data must meet few challenges including (i) reduction of the execution time, (ii) extraction of biologically relevant information and (iii) generation of different levels of information using the same tool. To effectively reduce the runtime, we have implemented few tricks. To reduce the network size as well as to retain biologically significant information as much as possible, No3CoGP first determines the network density cut-off value from the initial reference co-expression network. Based on this cut-off value, the co-expressed gene pairs have been filtered to construct a new network for further analysis. Moreover, the flexibility lies in the setting of parameters according to the depth of the study. The users can choose other two filtering options. Briefly, one is the selection of gene pairs having higher *r*-values and another is the selection of statistically significant *r*-values within two classes. Moreover, No3CoGP can utilize multiple CPU cores depending on the availability at user’s end. Our tool implements affymetrix annotation files and data files for a wide range of species to identify differentially co-expressed genes and gene modules. Utilizing biologically and statistically significant *r*-cutoff value for gene coexpression study it can identify different types of conserved and non-conserved genes and gene modules which are functionally significant.

Traditional differential expression analysis may not detect the changes in regulatory pattern of a gene. On the contrary, changes in the coexpression network structure may have predictive power to identify the candidate disease genes [11]. To unravel the complex dysregulations, one must peep into the differentially coexpressed genes at a systems biology level [reviewed in [12]]. Choi et al. [2] observed correlation between increased (or decreased) coexpression interactions and enhancement (or inactivation) of functional interactions. However, an increase or decrease in the correlation of a gene pair may be due to the up- or down-regulation of other genes in the same functional category. The modular coexpression analysis can potentially highlight the novel disease-causing biomarkers. Here, the gene modules identified by IPCA algorithm would be useful to find out potential functions of the genes and also the genetic dysregulations among the classes. Further, the output files may be used to calculate various node topology statistics like the node degree, the betweenness, the closeness and other network properties using Cytoscape [13], Pajek [14], NeAT [15] etc. The user can visualize the graphs, compare those and also perform functional analysis.

### No3CoGP key functionalities

#### Data import

Affymetrix microarray data (.CEL files or log2 normalized.txt files) and the annotation files of interest have to be kept in the respective directories of the project. CDF file and probe file for each different project must be pre-installed. A text file containing the class names should be created and given as input. (See online documentation for details).

#### Probe mapping

Any one of the available annotation ids among ‘UniGene ID’, ‘Gene Symbol’, ‘Ensembl’ and ‘Entrez Gene’ can be used for analysis. Affy probeset ids will be mapped with their annotation ids. Probesets which will map to single id will only be retained.

#### Data preprocessing

User can use the raw microarray data or log2 normalized data as input. If raw data is used as input, either RMA or GCRMA of R-package may be used for data normalization. On the other hand, the above step will be skipped if log2 normalized data itself is provided as input. When multiple probes map to the same gene and if the expression values of these probes vary in a particular condition, we have followed the method used by Dozmorov et al. [16] to set the expression value of that gene. Let us consider a case that the three probes (P1, P2 and P3) map to the gene G1 and we are considering expression of the gene G1 in two different samples (Sample 1 and Sample 2). Further we consider that the expression of these three probes vary within a sample. If the expression of probe P1 is highest in Sample 1, then the expression of the gene G1 is set to the expression value of the probe P1. On the other hand, if the expression of probe P3 is highest in Sample 2, then we set the expression of gene G1 to the value of expression of probe P3 in Sample 2.

#### Filtering based on coexpression values

Coexpression values of gene pairs will be calculated using Pearson Correlation Coefficient (*r*) or Spearman’s rank Correlation Coefficient (*ρ*). The formulae are as follows  where r = Pearson correlation coefficient, *x*_*i*_ = values in first set of data, *y*_*i*_ = values in second set of data and n = total number of values and  where *ρ* = Spearman’s rank correlation coefficient, n = number of paired ranks and *d*_*i*_ = difference between the paired ranks. Considering genes as nodes and giving a link between them, if |*r*| > 0.6, a coexpression network will be constructed [10, 17, 18]. The density (D) of the network for each *r* value will be calculated according to the formula D=2E/K(K-1), where E is the number of actual links and K is the number of non singleton nodes. To extract useful information from gene coexpression data, we have followed the method described in [10]. Thus, the gene pairs which satisfy |*r*| > *r*_*bs*_ will only be considered in next steps. The *r*_*bs*_ is the *r*-value where the network density of the coexpression network is minimum. User, further, can choose statistically significant (any of top 1%, 5%, 10%, 50% or all) gene pairs (from those with |*r*| > 0.6) for their analysis. The *P*-values of *r*-values have been determined by calculating t statistic using  where d=degrees of freedom. Its significance level *P*, is given by Student’s Distribution Probability Function [19]: *P* = *A*(*t*|*d*).

#### Identification of conserved and non-conserved gene pairs

A gene pair is defined as conserved if it is coexpressed in both the classes with same sign. It can be classified into two types (i) PCCGP (if *r*-value is positive) and (ii) NCCGP (if *r*-value is negative). On the contrary, the gene pair which is coexpressed only in one class, or coexpressed in both the classes but with different signs is defined as non-conserved gene pair. Those are termed as DCNCGP or CCNCGP, respectively. When the coexpression values of a gene pair in two classes will be compared, only the gene pair having significantly different *r*-values in two classes will be considered. Gene pairs will be identified at the user defined significance level (1%, 5% or 100%) by Fisher’s z-test [18]. If there are two correlations with sample sizes *n*_1_ and *n*_2_, both of these are transformed into Fisher’s Z values, . Under the null hypothesis that the population correlations are equal, the Z value, , has an approximately normal distribution.

#### Identification of modules

The gene modules will be identified using IPCA algorithm [20] which uses a combination of subgraph diameter and subgraph density. Li et al. [20] showed that the algorithm IPCA works better to identify complexes than previously proposed clustering algorithms, including DPClus, CFinder, LCMA, MCODE, RNSC and STM. IPCA algorithm requires a threshold (*T*_*in*_ ranging between 0 and 1) and a diameter (*d*) which is a positive integer.

#### Results output

Following the dialog windows, users can obtain a full list of gene pairs coexpressed in a specific class; positively, negatively or contra-coexpressed gene pairs, their *r*-values and *P*-values. The result files will be stored in respective subdirectories of ‘Output’ directory under user-defined project. Similarly, information of the gene modules will be saved in two different files under the respective ‘Modules’ sub-directories. One of them contains module number along with its participating genes; the other contains two column data (related gene pairs) which can be utilized in other network analyzing software like Cytoscape [13], Pajek [14], NeAT [15] etc.

### Conclusion

Starting from the Affymetrix raw data or log2 normalized data, No3CoGP provides a user friendly platform to identify both differentially conserved and non-conserved gene pairs and to generate the modules of genes. Within the conserved category, user can get positively and negatively coexpressed gene pairs separately. Again, within the non-conserved category, it can classify the contra-coexpressed gene pairs and gene pairs which are coexpressed in only one class. Using up-to-date annotation files provided by the users and biologically and statistically significant *r*-value as cutoff identified by the tool itself, No3CoGP expedites extracting useful information. It can utilize single or multiple CPU cores depending on the availability. The output files stored in the respective directories can be used for further analysis.

## Availability and requirements

**Project name:** No3CoGP

**Project home page:**http://www.bioinformatics.org/no3cogp/

**Operating system(s):** Linux/Unix, Windows (32 bit and 64 bit)

**Programming language:** Java

**Other requirements:** Java JRE 1.7 or higher, R, Bioconductor packages (see online documentation)

**License:** NA

**Any restrictions to use by non-academics:** None.

## Availability of supporting data

Sample data: http://www.bioinformatics.org/no3cogp/downloads/sample_data.zip

Additional file: http://www.bioinformatics.org/no3cogp/downloads/additional_file.xls

## Electronic supplementary material

Additional file 1: **Coexpressed gene pairs and modules.** The.xls file contains results of test data of tomato. Index sheet describes other eight different sheets. (XLS 10 MB)

## Abbreviations

CDF: Chip description file
GUI: Graphical user interface
CPU: Central processing unit.
